# Unlocking Carbon Emissions and Total Factor Productivity Nexus: Causal Moderation of Ownership Structures via Entropy Methods in Chinese Enterprises †

**DOI:** 10.3390/e27101048

**Published:** 2025-10-09

**Authors:** Ruize Cai, Jie You, Minho Kim

**Affiliations:** 1 Department of International Trade, Jeonbuk National University, Jeonju 54896, Republic of Korea; 2 School of Information and Software Engineering, East China Jiaotong University, Nanchang 330013, China

**Keywords:** carbon emissions reduction, ownership structure, total factor productivity, entropy

## Abstract

Amidst global imperatives for environmental sustainability, this study investigates the nexus between carbon emissions reduction (CER), ownership structures, and total factor productivity (TFP) in Chinese enterprises—recognized as vital economic drivers facing carbon emissions pressures. Based on the theoretical frameworks of innovation offsets, agency cost theory, and upper echelons theory, with data from CSMAR (2009–2023), we proposed a positive effect of CER on TFP while examining the moderating roles of ownership structure metrics: chairman shareholding ratio, manager shareholding ratio, and ownership–control separation ratio. TFP estimation employed dual approaches: mean consolidation (TFP-Mean) and entropy weighting (TFP-Entropy) methods. The results confirmed CER exerts significantly positive impacts on TFP, with ownership structures demonstrating statistically significant yet directionally heterogeneous moderation effects. Heterogeneity analysis reveals heightened TFP sensitivity to carbon emission initiatives among private enterprises, foreign-owned enterprises, and small enterprises. Notably, the entropy weighting method exhibits substantial comparative advantages in TFP measurement. These findings underscore that advancing TFP necessitates simultaneously optimizing carbon emissions efficiency and ownership governance.

## 1. Introduction

Carbon emissions reduction (CER) and corporate sustainable development have emerged as pivotal global issues. With the deepening implementation of China’s “dual-carbon” policy (carbon peaking and carbon neutrality), enterprises face increasingly stringent emission reduction pressures. While pursuing carbon reduction and sustainable development strategies, Chinese enterprises should simultaneously prioritize enhancing total factor productivity (TFP) to facilitate industrial upgrading and productivity advancement. International research reveals a complex relationship between CER and TFP: The Porter Hypothesis posits that appropriate environmental regulations may boost productivity through innovation compensation effects [1], while the Environmental Kuznets Curve (EKC) theory suggests a potential inverted U-shaped relationship [2]. Additionally, underlying nonlinear relationships exist among total factor productivity, energy consumption, and carbon emissions, alongside bidirectional causal linkages between these variables [3]. However, consensus remains elusive regarding the precise mechanisms driving corporate decarbonization in China’s unique institutional background [4]. Constrained by developmental lag, Chinese enterprises lack a systematic understanding of synergistic pathways between emission reduction and TFP, particularly given data deficiencies and dynamic ownership structure evolution. The mechanism through which carbon reduction affects TFP via corporate governance requires in-depth investigation. This study not only addresses a critical gap in understanding how governance structures reshape the efficiency frontier of CER-to-TFP conversion in Chinese enterprises, but also specifically examines the synergistic governance effects arising from Chairman and manager shareholding under conditions of ownership–control separation.

As pivotal engines of national economic growth, China’s listed enterprises play a crucial role in driving social economic development. Their low-carbon transition is essential for achieving dual-carbon targets. Increasing global emphasis on carbon emissions—driven by governmental regulations and corporate ESG commitments—compels management to consider how environmental performance impacts corporate value, profitability, and TFP [5]. Furthermore, proactively propelling innovation in corporate production technologies synergistically promotes both internationalization progress and corporate performance [6]. Particularly for Chinese enterprises actively expanding global markets through internationalization, balancing emission reduction imperatives with productivity enhancement becomes operationally critical. This study addresses three research gaps through quantitative empirical analysis integrating multi-source emissions data: (1) whether CER improves corporate TFP; (2) how ownership structures moderate the carbon-TFP relationship; and (3) methodologically innovating by applying the entropy weight method to quantify and integrate TFP and ownership structure variables into micro-level firm empirical analysis.

To address these questions, we employ systematic methodologies with a comprehensive literature review. Using data from the China Stock Market and Accounting Research (CSMAR) database, we construct a composite carbon reduction index and ownership structure variables via entropy weighting. Our empirical analysis proceeds in two stages: First, examining the carbon-TFP relationship; second, verifying the ownership structure’s moderating effect via three innovative dimensions: chairman shareholding ratio, CEO shareholding ratio, and the separation ratio of ownership and control [7]. Methodologically, we apply pooled OLS regressions and heterogeneity analysis, Heckman selection models to correct sampling bias, regression discontinuity design (RDD), and differences-in-differences (DID) for policy shock identification. The results demonstrate (1) a significant positive impact of carbon reduction on TFP; (2) stronger moderating effects from chairman/manager shareholdings than other ownership metrics; (3) superior explanatory power of entropy-weighted TFP (*TFP-Entropy*) over mean-aggregated TFP (*TFP-Mean*) derived from five complementary measurement approaches (OLS, FE, LP, OP, and GMM); (4) pronounced effects in private-owned enterprises (POEs) and large firms.

This study makes three primary contributions to the literature. First, we empirically confirm that corporate responses to carbon reduction measures exert a significantly positive impact on TFP in China. While prior studies have provided partial insights into carbon emissions’ effects in the Chinese context, consensus remains elusive regarding both the direction and underlying mechanisms of the carbon-TFP relationship [8]. Our findings on the productivity-enhancing effects of carbon reduction reconcile the theoretical tension between the emission abatement cost hypothesis [9] and the innovation compensation theory [10], thereby providing micro-level evidence for synergistic productivity gains under the dual-carbon policy framework.

Second, our research establishes ownership structure as a pivotal moderator in the carbon–TFP relationship. Departing from conventional analyses that predominantly examine ownership types [11], we innovatively deconstruct its structural dimensions into three distinct governance mechanisms: chairman shareholding ratio, manager shareholding ratio, and the separation ratio between ownership and control rights. This dimensional approach reveals a significant difference in moderating effects: while chairman and manager shareholdings amplify carbon reduction’s productivity gains. These differential patterns extend the upper echelons theory [12] into environmental performance research by demonstrating how firm-specific power configurations shape strategic responses to sustainability pressures—a previously underexplored theoretical linkage.

Third, methodologically, we pioneer the application of the entropy weighting method to quantify TFP and ownership structure variables. By leveraging information entropy for multi-dimensional aggregation, this approach overcomes the “simplification bias” inherent in conventional measures that rely on linear averaging or principal components. While the entropy weight method has been widely employed in macroeconomic research—for instance, Zhao et al. [13] utilized information entropy and industrial linkage to measure structural stickiness of TFP shocks, and Gao et al. [14] applied entropy to assess economic development and ecological construction levels—its application to firm-level studies remains limited. Although Chen et al. [15] discussed firm-level data, their use of entropy was confined to calculating the Digital–Green Synergy (GDS). Similarly, Albulescu et al. [16] focused solely on company-level data from German electricity and gas firms, employing a symbolic transfer entropy panel causality test. The use of entropy in analyzing ownership structure dates back to Jacquemin and Kumps [17], who proposed an entropy measure for firm size structure, arguing its superiority over the “overall concentration” approach. More recently, He et al. [18] adopted an entropy-TOPSIS-GRA evaluation model, noting that ownership structure affects financial performance in China’s electricity and gas sectors; however, their analysis was limited to comparing heterogeneity between state-owned and private-owned enterprises rather than developing an integrated aggregation methodology. Empirical validation confirms that entropy-weighted metrics exhibit superior robustness in capturing latent interactions and nonlinearities. In comparative analyses, entropy-derived TFP values reduce prediction errors versus mean-consolidated estimates, while ownership structure indices demonstrate enhanced resilience to threshold sensitivity. In contrast, this research extends the entropy approach to directly aggregate firm-level TFP and ownership structure variables, addressing a critical methodological gap in micro-empirical studies. This methodological advancement resolves critical limitations in traditional construct measurement.

The remainder of this paper proceeds as follows: Section 2 reviews the literature and develops testable hypotheses. Section 3 details data sources and variable construction (including entropy weighting procedures), followed by econometric methodology. Section 4 presents descriptive statistics and regression results, supplemented by robustness checks and causal identification analyses. Section 5 provides discussion, limitations, implications, and future research directions. Section 6 summarizes the conclusions.

## 2. Literature and Hypotheses

### 2.1. The Effect of CER Based on Innovation Offsets

The relationship between CER and TFP remains a central controversy in environmental economics. International studies grounded in the “innovation offsets” by Porter and Linde [1] contended that well-designed environmental regulations spur technological innovation, thereby enhancing resource allocation efficiency. Dechezleprêtre and Sato [19] demonstrated this mechanism in their analysis of the U.S. Clean Air Act, where regulated firms achieved TFP growth through energy efficiency improvements while simultaneously stimulating R&D innovation within affected industries. Furthermore, Wu et al. [20] suggested that technological innovation indirectly enhances environmental performance by improving operational efficiency. Distinct organizational resources and capabilities can lead to superior corporate ecological performance [21]. Meanwhile, the adoption of enterprise risk management (ERM) not only contributes to enhancing organizational legitimacy and fostering positive stakeholder relationships but also promotes green growth within organizations [22]. However, scholars such as Gray and Shadbegian [9] presented counterevidence: their study of the U.S. paper industry reveals that environmental compliance costs directly crowd out productive investment, leading to measurable TFP declines. This critique is further reinforced by Acemoglu et al. [23], whose endogenous growth model indicates that when clean technologies yield lower returns than conventional alternatives, emission reduction triggers “transitional productivity losses”. Additionally, Cainelli et al. [24] observed that environmental motivation exerts an insignificant or even negative impact on labor productivity growth in specific service sectors in the short term, which appears inconsistent with the Porter effect. Consequently, the drivers of capital efficiency and eco-efficiency may not always be entirely aligned [25].

China, as the world’s largest carbon emitter, exhibits unique complexities in the CER-TFP relationship. On one hand, heterogeneous policy-industry interactions yield divergent outcomes. Cheng and Meng [26] demonstrated that China’s carbon emissions trading policy suppresses listed firms’ TFP primarily due to heightened compliance costs. In contrast, Wu and Wang [27] identified a persistent positive causal relationship between carbon emission prices and TFP, mediated by increased investments in R&D patents and financing. Sectoral analyses further reveal differential impacts. Ge et al. [28] confirmed that environmental regulations enhance manufacturing (secondary sector) productivity while inhibiting TFP in agriculture (primary sector) and services (tertiary sector). Yet counterevidence emerges from Pan et al. [29] and Yang and Zhang [30], who documented significant TFP gains in carbon trading pilot firms through market-based incentives. This dichotomy is ultimately reconciled by Cheng and Kong [31], whose integrated environmental policy portfolio framework demonstrates that technical innovation enables environmental regulations to stimulate green TFP growth. The observed divergence is attributable to policy instrument designs: administrative mandates tend to trigger compliance costs, whereas market-based instruments more effectively induce innovation and resource reallocation [28]. On the other hand, firm-specific technological capacity constitutes a critical threshold. Li and Cheng [32] revealed that technology-intensive manufacturers in China achieve higher total-factor carbon efficiency, while Cheng and Kong [31] and Wang et al. [33] jointly established that technological progress and industrial structure upgrading accelerate TFP growth. These patterns align with European evidence where emissions trading systems stimulate low-carbon innovation and productivity in frontier firms [34,35], though China’s transformation likely incurs heightened transition costs.

Current research exhibits three critical gaps. First, endogeneity concerns in the CER-TFP relationship remain inadequately addressed. Hasanov et al. [36] posited that high-TFP firms may proactively reduce emissions, suggesting potential reverse causality rather than CER driving productivity gains. Mukhtarov [37] further identified concurrent negative impacts on carbon emissions from TFP, renewable energy adoption, and export activities. Zhong and Wang [38] demonstrated an inverted U-shaped curve linking forestry TFP to carbon emissions, while Yang et al. [39] confirmed that industrial structure upgrading and green TFP development jointly suppress carbon emissions. Second, insufficient attention is paid to heterogeneity across firm sizes and industries, with extant studies lacking rigorous causal identification strategies. For instance, Liu et al. [40] merely discussed sectoral and ownership-type variations. Third, conventional TFP measurement approaches fail to capture the technological essence of CER impacts. Li and Li [41] attributed this to inherent methodological flaws in traditional techniques, which generate inconsistent estimates and underscore the imperative of selecting appropriate productivity metrics. This study’s Hypothesis 1 aims to transcend these limitations by leveraging Chinese firm-level microdata to verify the causal effect of CER on TFP, thereby revealing boundary conditions for Porter Hypothesis applicability across heterogeneous enterprises under alternative measurement frameworks. Based on the theory of innovation offsets within the Porter Hypothesis, we propose the following first hypothesis:

**Hypothesis** **1.** *CER is positively related to TFP in Chinese firms.*

### 2.2. Moderating Effect of Ownership Structures Based on Agency Cost Theory and Upper Echelons Theory

The moderating role of ownership structures in the CER-TFP relationship is emerging as a frontier in interdisciplinary research bridging corporate governance and environmental economics. International scholarship primarily advances along two theoretical paths: agency cost theory and upper echelons theory. First, building on the agency cost theory by Jensen and Meckling [7] and Ang et al. [42] ownership concentration is shown to shape long-term strategic orientation. Azimi et al. [43] confirmed that the ownership structure exerts nonlinear effects on aggregate enterprise productivity growth. Complementary evidence from Ben-Amar and André [44] indicated that dispersed ownership structures entail lower agency costs without detrimental performance impacts. Hill and Snell [45] further established that ownership configurations directly and indirectly influence productivity through diversification strategies, R&D expenditures, and capital intensity. Second, leveraging Hambrick and Mason [12]’s upper echelons theory, Shahab et al. [46] demonstrated that executive characteristics filter environmental decisions through cognitive lenses. However, the development of agency theory has not been limited to this perspective; incentive compatibility mechanisms also create possibilities for achieving organizational objectives [47]. Modern corporate governance mechanisms are designed to reduce agency costs and align managers’ interests with the long-term value of shareholders. Consequently, a revised agency perspective centered on incentive compatibility has emerged [48]. Hindley [49] observed divergent managerial behaviors following ownership-management transitions, while Cheon et al. [50] revealed that ownership restructuring enhances TFP. Mnasri and Ellouze [51] confirmed the pivotal role of family-controlled firms in boosting productivity, thereby strengthening the CER-TFP relationship. Contrastingly, Wang et al. [52] cautioned that excessive fiscal decentralization among shareholders heightens resistance to environmental regulation implementation. Furthermore, Shahrour et al. [53] suggested that higher CEO ownership is associated with lower commitment to carbon reduction goals, yet adversely influences carbon disclosure decisions [54]. Claessens et al. [55] specifically documented that the separation of ownership and control proves most pronounced in family-controlled enterprises and smaller firms within Asian economies.

The moderating mechanism of ownership structures in Chinese enterprises exhibits a unique three-dimensional characterization: chairman shareholding ratio, manager shareholding ratio, and the separation ratio between ownership and control rights. While international research has addressed the generic role of ownership concentration [56,57], analyses remain insufficient regarding the distinctive corporate governance context in China. Focusing on the Chairman’s shareholding dimension, Lu et al. [58] revealed that power centralization may elevate decision-making risks but simultaneously enhances procedural efficiency. Complementary evidence from Chiang and Lin [59] indicated that smaller boards experience fewer bureaucratic impediments, thereby improving functional productivity. Managers shareholding restructures managerial behavior through incentive alignment and executive characteristics. Bulan et al. [60] confirmed that CEO’s emolument sensitivity to stock options relates with productivity positively. However, Shahrour, Arouri, Tran and Rao [53] demonstrated that CEOs with substantial ownership stakes (5–10%) exhibit the strongest propensity to deprioritize CER. Contrastingly, Sun et al. [61] established that equity incentives positively moderate the relationship between CEO stability, TFP, and green innovation. Shen [62] further identified that CEOs’ overseas experience enhances the TFP-boosting effect of ESG performance. Nevertheless, these effects are significantly constrained by ownership–control separation in Chinese firms. Current empirical research on control rights’ efficiency implications faces methodological challenges due to complex ownership patterns [63]. Boubaker et al. [64] substantiated that excessive control rights suppress production efficiency, while Kim and Na [65] observed improved social-environmental ratings when ownership–control divergence widens post-merger. Sun et al. [66] documented positive associations between family ownership/control and ESG scores, though market competition negatively moderates this relationship.

Existing research exhibits three critical limitations. First, ownership structure is conducted in relatively isolated dimensions. Most literature exclusively examines isolated ownership indicators—primarily focusing on family holdings, largest holdings, and concentrated ownership [67,68,69]—while neglecting interactive effects between Chairman and manager shareholdings. When Chairman ownership is high, but manager ownership remains low, strategic decision–execution misalignment may undermine emission reduction efficiency. Second, moderating pathways remain ambiguous. How ownership structure transmits corporate governance effects to emission technology choices (e.g., end-of-pipe treatment vs. source innovation) has yet to be clarified. While Alfi et al. [70] identified a positive relation between board size and carbon disclosure. Elsayih et al. [71] observed higher carbon performance in firms with greater board independence. Third, China’s corporate governance transition remains inadequately integrated with carbon reduction dynamics, despite evidence linking managerial ownership to carbon disclosure transparency [72]. Although ownership–control separation typically intensifies post-IPO in private enterprises (e.g., through founder divestment diluting control rights), no study has dynamically analyzed its evolving moderating mechanisms in emission reduction [73,74]. This revised agency perspective, termed “incentive compatibility”, posits that external pressures can reshape the incentive structure under significant separation of ownership and control, thereby aligning the controlling shareholder’s self-interest with the firm’s long-term green transition goals [75]. Under strong external institutional pressures—such as stringent environmental regulations, intense green market competition, and strong public environmental demands—controlling shareholders in firms with high ownership–control separation may recognize that strong environmental performance is crucial for maintaining corporate legitimacy, obtaining policy benefits, avoiding penalties, and preserving reputational capital related to control [76]. In such contexts, to protect and maximize their private benefits of control, they may have strong incentives to urge management to effectively implement carbon emission reduction projects and ensure these are genuinely transformed into productivity gains and competitive advantages [77]. Under these conditions, a high degree of ownership–control separation may enhance the positive effect of CER on TFP. The innovative value of Hypothesis H2 lies in constructing an integrated model addressing China’s tripartite ownership structures characteristics: Chairman shareholding ratio reflects strategic decision-making resolve (overcoming short-termism), manager shareholding ratio represents executive incentive intensity (technology implementation efficiency), and ownership–control separation signifies agency conflict magnitude (resource misallocation risks). This framework transcends conventional unidimensional moderation models by revealing synergistic governance logic within China’s transitional economy, thereby providing theoretical foundations for aligning environmental policies with corporate governance structures. Consequently, drawing on agency cost theory and upper echelons theory, we develop a set of moderated relationships. Informed by Baron and Kenny [78]’s multidimensional approach to identifying moderator attributes, we propose one comprehensive moderator followed by three specific moderated hypotheses. Building on this foundation, we further advance a second comprehensive hypothesis that integrates both theoretical perspectives.

**Hypothesis** **2.** *Ownership structures play a moderating role in the relationship between CER and TFP.*

For the first specific moderator, grounded in agency cost theory, we examine the governance and environmental implications when ownership is highly concentrated in the chairman.

**Hypothesis** **2a.** *In ownership structures, chairman shareholding negatively moderates the relationship between CER and TFP.*

For the second moderator, based on the upper echelons theory, we contend that executive characteristics shape strategic decisions and performance outcomes.

**Hypothesis** **2b.** *In ownership structures, manager shareholding positively moderates the relationship between CER and TFP.*

From the perspectives of agency cost theory and the lens of incentive compatibility, we propose a third specific moderator, suggesting that effective supervisory and incentive mechanisms can mitigate the adverse effects associated with the separation of ownership and control.

**Hypothesis** **2c.** *In ownership structures, ownership–control separation positively moderates the relationship between CER and TFP.*

## 3. Data and Methodology

### 3.1. Sample and Data

This study collected annual data from companies listed on the Shanghai and Shenzhen Stock Exchanges between 2009 and 2023 as the research sample. All data were sourced from the China Stock Market and Accounting Research (CSMAR) Database and the Chinese Industrial Enterprises Database. Corporate carbon emission data and financial indicators were analyzed following a four-step data cleaning procedure: (1) excluding financial firms whose accounting information fails to reflect operational realities; (2) removing “ST” (Special Treatment) companies facing delisting risks; (3) eliminating observations with significant outliers or missing key variables; and (4) winsorizing continuous variables at the 1st and 99th percentiles. The final sample comprises 117 non-financial listed firms, yielding 1123 firm-year observations. Table 1 presents the descriptive statistics of key variables.

Furthermore, to ensure data continuity and mitigate the potential biases arising from singular or discontinuous data points—such as failure to form continuous variables or multicollinearity leading to non-convergence in parameter estimation—we acquired carbon emission-related data through multiple attempts and diverse channels, subsequently applying a mean imputation method. This approach involved calculating the average of actual values and imputing them into the adjacent years (t − 1 and t + 1), thereby enhancing the reliability of the model’s coefficient estimates. In this study, CER specifically refers to the reduction in CO_2_ and CO emissions (distinct from greenhouse gas emissions overall), measured in tons per year.

### 3.2. Variables

#### 3.2.1. Dependent Variables

The dependent variable is TFP, which captures output growth attributable to non-input factors such as technological progress, managerial optimization, and institutional innovation under given input conditions. This comprehensive metric reflects long-term development driven by productivity enhancements and industrial upgrading. TFP is conventionally estimated through two primary methodologies: growth accounting and production frontier approaches. The growth accounting method, often applied in macroeconomic aggregate analyses due to its simplicity and historical comparability, remains a widely utilized tool. In contrast, the production frontier approach is more appropriate for investigating the roots of efficiency loss or for comparing efficiency across micro-level entities, as it enables the identification of items of technical inefficiencies and permits decomposition analyses. Accordingly, we collected TFP estimates for listed companies computed via five standard econometric techniques—OLS, FE, LP, OP, and GMM. These methods aim to accurately “strip out” contributions from capital, labor, and other conventional inputs from total output, with the residual representing TFP. These residual captures growth components unexplained by traditional factors, such as technological progress and efficiency enhancements. These estimates were then integrated using both the mean-value method and entropy weight method to derive two composite TFP measures: *TFP-Mean* and *TFP-Entropy*. These composite indicators serve as our primary TFP metrics for comparative empirical analysis.

#### 3.2.2. Independent Variables

The independent variable, *CER*, is defined as the decrease in carbon emissions. The attained reduction in carbon emissions results from enterprises implementing low-carbon technologies and optimizing energy structures within their operational practices. While discharging carbon emission reduction responsibilities, enterprises can simultaneously capture carbon asset revenues and enhance core competitiveness through elevated ESG ratings. This firm-level annual Δ*CER* metric serves as the core independent variable in our empirical models. Drawing upon the CSMAR database’s definition of carbon emission reduction—namely, Δ*CER* = Emission_t−1_−Emission_t_—we directly cleaned, imputed, and incorporated the obtained carbon emission reduction data into regression analyses. Nevertheless, relying solely on first differences may inadequately address confounding influences from business cycles, output variations, or scale effects, which constitutes a limitation of this research. To minimize potential biases, we also controlled for variables including enterprise assets, profitability, and leverage ratio. Future studies should consider adopting standardized measures of carbon emission reduction, such as carbon intensity per unit of output, to better isolate genuine efficiency improvements.

#### 3.2.3. Moderating Variables

The moderating variable is ownership structure, which is conventionally categorized into ownership composition and ownership concentration. Consequently, this study employs three related metrics—chairman shareholding ratio, manager shareholding ratio, and ownership–control separation ratio—to reshape the pathway through which CER influences TFP. The chairman’s shareholding ratio reflects the centralization of strategic decision-making authority; elevated levels may curb short-termism tendencies. The manager’s shareholding ratio quantifies the alignment degree between executive authority and residual claim rights, thereby affecting the implementation efficacy of emission-reduction technologies. The ownership–control separation ratio measures deviations in control rights allocation, where agency conflicts may induce misallocation of emission-reduction resources. Collectively, these three-dimensional characteristics constitute a “decision mechanism” spanning governance intention, execution incentives, and agency costs. This framework reveals the mechanism by which heterogeneous governance structures moderate TFP impacts, thereby capturing complexities of China’s corporate governance transition more effectively than singular ownership concentration metrics.

#### 3.2.4. Control Variables

This study controlled for a set of firm-specific characteristics and financial conditions grounded in established corporate finance and industrial organization theories, which are hypothesized to potentially influence both the dependent variable and the key explanatory variables [79], thereby mitigating omitted variable bias and sharpening the identification of the net effect of our primary variables of interest [80]. Specifically, we incorporated listing age (*Age*), total assets (*TAssets*), R&D expense (*RDExp*), operating revenue (*OReve*), number of employees (*Employee*), and total leverage ratio (*Leve*) as control variables. The selected controls were chosen for their theoretical relevance in capturing fundamental dimensions of firm size, resource endowment, innovation capacity, operational scale, and financial structure [81,82]. Their inclusion aims to isolate the core relationship under investigation by accounting for potential confounding variations, even if some controls are not statistically significant in the specific sample, as their primary role is to enhance the internal validity of the model rather than to be interpretable for their independent effect [83]. Detailed definitions and measurements for all variables are comprehensively provided in Appendix A to ensure transparency and reproducibility.

### 3.3. Model Selection

In the model selection process involving pooled OLS, fixed effects (FEs), and random effects (REs) specifications, the F-test and Hausman test are conventionally utilized to determine the most appropriate estimator. The F-test initially examines whether significant differences exist in coefficient estimates between the pooled OLS and FE models. If the null hypothesis is rejected, the Hausman test is subsequently employed to evaluate whether the fixed effects model outperforms the random effects alternative; otherwise, the pooled OLS model is retained. The significance level of the F-statistic denotes the probability of committing a Type I error, which occurs when the null hypothesis is incorrectly rejected. In this study, the F-test results did not lead to rejection of the null hypothesis at the 1% significance level. Additionally, the pooled OLS model assigns equal weight to within-group deviation, representing variation within each individual over time, and between-group deviation, which captures differences across distinct individuals. The FE model exclusively accounts for within-group deviation, thereby eliminating the influence of time-invariant individual characteristics on regression estimates. In contrast, the RE model is analogous to generalized least squares (GLS) regression, yielding estimates that constitute a weighted average of the within-group and between-group estimators, with the weight of the between-group component ranging from 0 to 1, thereby positioning the result between the pooled OLS and FE outcomes. After integrating theoretical mechanisms, model assumptions, and statistical test findings, the pooled OLS model was ultimately selected for regression estimation, a decision that contributes to reducing potential estimation bias.

In addition, this study initially did not incorporate two-way fixed effects due to two primary objective constraints. First, the sample covers a relatively long time span, and a significant number of firms exhibit extensive missing values across different years. Introducing two-way fixed effects in such a panel dataset would excessively consume degrees of freedom, severely reducing estimation precision or even preventing model convergence. Second, the key explanatory variables demonstrate minimal variation over time, with most of their variance existing between entities. Including entity fixed effects would absorb this cross-sectional variation, thereby negating the substantive interpretation of the estimates. To address potential methodological concerns, we conducted extensive robustness checks and endogeneity tests. The core findings remain qualitatively consistent across these alternative specifications. We acknowledge this limitation and intend to address it in future research when more comprehensive data become available. Furthermore, the presence of numerous redundant variables in the tables necessitated a streamlined presentation of key and control variables to enhance readability. In empirical research, the primary purpose of including control variables is to isolate the net effect of key explanatory variables by holding potential confounding factors constant, thereby ensuring unbiased coefficient estimates for key variables such as CER and ownership structure. The selection of these controls was grounded in solid theoretical foundations and prior literature, rather than arbitrary addition. Therefore, although certain control variables did not achieve statistical significance within this specific sample, this outcome may be attributed to sample-specific characteristics or inherent data limitations. Future investigations could further explore the influence of these variables across different contexts or improved datasets.

### 3.4. Model Design

This study employed financial and carbon emission data to test Hypotheses 1, 2, 2a, 2b, and 2c using pooled OLS regression models with interaction terms. First, we analyzed the relationship between CER and TFP using a pooled OLS model. Subsequently, we examined the moderating effects on the CER-TFP relationship by sequentially incorporating three ownership structure moderators—chairman shareholding ratio (*CShare*), manager shareholding ratio (*MShare*), and ownership–control separation ratio (*Separation*)—as interaction terms with *CER*. Robustness test were conducted using alternative TFP measurements derived from different estimation methodologies.

The regression specifications are formalized below. Equation (1) tests Hypothesis 1:

(1) $${TFP}_{it}=\alpha_{0}+\alpha_{1}{CER}_{it}+\alpha_{2}{Controls}_{it}+\varepsilon$$

where *TFP_it_* denotes the dependent variable for firm *i* in year *t*, *CER_it_* represents the independent variable, *Controls_it_* is the control variables, *α_0_* is the intercept, *α_1_* and *α_2_* are coefficients, and *ε_it_* is the error term. A significantly positive *α_1_* (*p* < 0.05) supports a positive moderating effect of CER on TFP.

Equations (2)–(6) test Hypothesis 2, 2a, 2b, and 2c by adding the following interaction terms:

(2) $${TFP}_{it}=\alpha_{0}+\alpha_{1}{CER}_{it}+{\alpha_{2}OwnStrEnt}_{it}+\;\alpha_{4}{Controls}_{it}+\varepsilon_{it}$$

(3) $${TFP}_{it}=\alpha_{0}+\alpha_{1}{CER}_{it}+{\alpha_{2}OwnStrEnt}_{it}+\alpha_{3}CER*{OwnStrEnt}_{it}+\;\alpha_{4}{Controls}_{it}+\varepsilon_{it}$$

(4) $${TFP}_{it}=\alpha_{0}+\alpha_{1}{CER}_{it}+{\alpha_{2}CShare}_{it}+\alpha_{3}CER*{CShare}_{it}+\;\alpha_{4}{Controls}_{it}+\varepsilon_{it}$$

(5) $${TFP}_{it}=\alpha_{0}+\alpha_{1}{CER}_{it}+{\alpha_{2}MShare}_{it}+\alpha_{3}CER*{MShare}_{it}+\;\alpha_{4}{Controls}_{it}+\varepsilon_{it}$$

(6) $${TFP}_{it}=\alpha_{0}+\alpha_{1}{CER}_{it}+{\alpha_{2}Separation}_{it}+\alpha_{3}CER*{Separation}_{it}+\;\alpha_{4}{Controls}_{it}+\varepsilon_{it}$$

where moderator variables denote *OwnStrEnt*, *CShare*, *MShare*, or *Separation*. A statistically significant *α_3_* (*p* < 0.05) confirms the moderating role of ownership structure in the CER-TFP relationship.

### 3.5. Comparison of Entropy Weight Methods

The entropy weight method represents an objective weighting approach wherein the assigned weights are determined by the information entropy inherent in the data itself. A higher degree of dispersion in indicator values (corresponding to lower entropy) yields a greater weight, which enables the method to effectively capture the differential contributions of various TFP estimation techniques, such as OLS, FE, LP, OP, and GMM. This stands in fundamental contrast to principal component analysis (PCA) and similar methods designed to extract the maximum variance and identify the principal directions within datasets. While orthogonal transformations in PCA may lead to the loss of certain informational features, the entropy weight method preserves the independent information from each constituent method. Its purported advantage in reducing simplification bias originates from this characteristic: it avoids discarding heterogeneous information that might represent unique mechanisms merely for the purpose of dimensionality reduction.

Empirically, we performed identical regression analyses using both mean-aggregated TFP (*TFP-Mean*) and entropy-weighted aggregated TFP (*TFP-Entropy*) as dependent variables. Based on the comparison of goodness of fit (R^2^) between the *TFP-Mean* and *TFP-Entropy* models, along with the statistical significance of key variable coefficients, the *TFP-Entropy* model demonstrates greater consistency with theoretical expectations. Collectively, these outcomes validate the effectiveness of employing the entropy weight method. To enhance the persuasiveness of our findings, we systematically evaluated the reliability of the entropy weight method used for index construction through multiple robustness assessments. These tests included adopting alternative variable measurements, performing sample adjustments (e.g., excluding specific industries or outliers), modifying model specifications, conducting heterogeneity analyses based on ownership structure and enterprise size, and addressing endogeneity using econometric models. Therefore, the multiple robustness tests confirm that the core conclusions remain statistically unchanged, indicating that the composite index constructed via the entropy weight method is reasonably robust. Furthermore, the weights for each TFP estimation method were calculated using the entropy weight method, which determines weight values based on the dispersion degree (information entropy) of each indicator dataset; greater dispersion results in a higher weight assignment. The weighting procedure involved data standardization, entropy calculation, computation of deviation coefficients, and final normalization. The weights for the five TFP methods are all positive and sum to one. It should be noted that weights were calculated by year group, resulting in minor annual fluctuations. The average weights over the entire sample period are as follows: OLS method ≈ 0.21, FE method ≈ 0.19, LP method ≈ 0.23, OP method ≈ 0.18, and GMM method ≈ 0.19. The weight calculation is entirely based on the intrinsic characteristics of the data, highlighting the relative importance of each method in terms of information contribution.

## 4. Empirical Results

### 4.1. Descriptive Statistics

Table 1 reports essential descriptive statistics for all key variables. Despite the extensive availability of corporate financial disclosures, carbon emission data remain comparatively limited due to collection constraints. Results indicate that *TFP-Mean* values ranged from 5.8 to 11.2 across firms, with a mean of 8.027. *TFP-Entropy* exhibited a distribution spanning 0 to 1, averaging 0.406. The mean *CER* value of 9.149 reflects insufficient corporate emission reduction intensity. The average firm listing age of 9.452 years suggests long listing periods and stabilized production operations among sampled enterprises. Ownership type was operationalized as a dummy variable: state-owned enterprises (SOEs) = 1, privately owned enterprises = 2, and foreign-owned enterprises = 3. With an ownership type mean of 1.834, non-SOEs constitute the majority of the sample. Core metrics of Chinese listed companies demonstrate robust completeness, thereby meeting this study’s analytical requirements.

### 4.2. Baseline Regression of TFP-Mean

Table 2 presents basic empirical findings. Each column displays pooled OLS regression results with *TFP-Mean* as the dependent variable in baseline specifications. Column (1) reports estimates corresponding to Equation (1), yielding a *CER* coefficient (*α_1_*) of 0.037. Columns (2)–(4) present estimations for Equations (4)–(6) incorporating moderating variables sequentially. Results indicate *CER* coefficients of 0.045, 0.059, and 0.053 in columns (2)–(4), respectively, all statistically significant at the 5% level. This evidence demonstrates CER’s positive effect on corporate TFP, thereby validating Hypothesis 1. Regarding moderating effects, columns (2)–(4) report estimations integrating three ownership structure moderators. The interaction terms *CER* × *CShare*, *CER* × *MShare*, and *CER* × *Seperation* yield coefficients (*α_2_*) of −0.002, 0.003, and 0.001, respectively, each significant at the 10% level. These results confirm statistically significant moderating effects of ownership structures on the CER-TFP relationship. Specifically, column (2)’s *α_2_* coefficient yielded a negative value, denoting negative moderating effects. In contrast, columns (3) and (4) both produced positive *α_2_* coefficients, indicating positive moderating effects. Consequently, findings collectively support Hypotheses 2a, 2b, and 2c.

The positive and statistically significant coefficients of CER across all specifications (ranging from 0.037 to 0.059) indicate that a one-unit increase in carbon emission reduction is associated with an increase in *TFP-Mean* by approximately 0.037 to 0.059 units, ceteris paribus. In economic terms, this suggests that corporate investments in carbon emission reduction—often manifested in energy efficiency improvements, adoption of cleaner technologies, and process optimization—not only fulfill environmental responsibilities but also enhance resource allocation efficiency and technological innovation capabilities, thereby boosting overall productivity. The significantly negative coefficient for the *CER* × *CShare* interaction term (*α_2_* = −0.002) indicates that chairman shareholding negatively moderates the CER-TFP relationship. This suggests that in firms with highly concentrated ownership, decision-makers may prioritize retaining control rights and short-term financial performance over long-term environmental investments, possibly due to concerns about the immediate costs and oversight burdens associated with such initiatives. Conversely, the positive moderating effects of managerial shareholding (*CER* × *MShare*, *α_2_* = 0.003) and ownership–control separation (*CER* × *Seperation*, *α_2_* = 0.001) align with upper echelons theory and incentive compatibility predictions: when managers hold shares or experience separation between ownership and control, they are more incentivized to pursue long-term value creation through sustainable practices like CER, as these actions can enhance decision-making efficiency and performance outcomes, signaling superior governance to the market. This ultimately translates CER efforts into tangible productivity gains.

In the research design of this section, the primary objective of the core pooled OLS model was not to establish a strict causal relationship, but rather to initially reveal a benchmark regression pattern. As reported in Table 2, the baseline pooled OLS regression primarily aimed to preliminarily examine whether a robust statistical association exists between CER and TFP, thereby providing a foundational reference and initial evidence for subsequent more complex identification strategies. We fully recognized that pooled OLS estimators might produce biased results in the presence of reverse causality; therefore, the pooled OLS results were not used as the sole or core evidence supporting the causal claims of this study. The multiple methods subsequently implemented—including the Heckman selection model, RDD, and DID—are anticipated to substantially alleviate concerns regarding endogeneity, particularly reverse causality, thereby enhancing the credibility of the research conclusions. For instance, the Heckman selection model was employed to correct for potential biases arising from non-random sample selection. Meanwhile, the RDD approach, which leverages local randomization around a cutoff point of policy shock or a continuous variable, effectively identifies the local average treatment effect (LATE), thereby largely mitigating reverse causality issues caused by unobservable factors. Additionally, the DID model compares changes between treatment and control groups before and after a policy shock, effectively controlling for unobservable time-invariant individual characteristics and common time trends, thus providing stronger support for causal inference. These models address endogeneity challenges from different perspectives, collectively strengthening the robustness of the conclusion that CER exerts a positive influence on TFP.

### 4.3. Baseline Regression of TFP-Entropy

Table 3 presents key empirical outcomes featuring pooled OLS regressions with *TFP-Entropy* as the dependent variable, distinguishing this analysis from prior specifications. Column (1) reports Equation (1) estimates, yielding a *CER* coefficient (*α_1_*) of 0.005. Columns (2)–(4) present moderated regressions per Equations (4)–(6), with *CER* coefficients registering 0.002, 0.004, and 0.004, respectively. Statistical significance at the 5% level was observed selectively, providing partial support for CER’s positive influence on corporate TFP. Moderating effect analyses reveal: Columns (2)–(4) incorporate three ownership structure interactions. Coefficients for *CER* × *CShare*, *CER* × *MShare*, and *CER* × *Seperation* (*α_2_*) are −0.001, 0.002, and 0.005, respectively, each significant at the 10% level. These results confirm significant moderation effects across ownership structures. Specifically, column (2)’s negative *α_2_* contrasts with positive coefficients in columns (3)–(4). Consequently, findings collectively validate Hypotheses 2a, 2b, and 2c.

The generally positive *CER* coefficients (ranging from 0.002 to 0.005), albeit with selective significance, still indicate a positive association between carbon emission reduction efforts and the composite TFP measure (*TFP-Entropy*). The smaller magnitude of coefficients compared to the *TFP-Mean* results may stem from the entropy method’s inherent weighting mechanism, which assigns weights based on the discriminant power of each underlying TFP estimator (OLS, FE, LP, OP, and GMM) and year group, potentially placing less emphasis on estimators that are more sensitive to *CER* changes. Economically, this reinforces the notion that the productivity benefits of CER are robust across different measurement methodologies, though the effect size might vary. The consistency in the signs and significance of the ownership structure moderators with the *TFP-Mean* analysis (e.g., *CER* × *CShare* negative, *CER* × *MShare* and *CER* × *Seperation* positive) underscores the fundamental role of corporate governance in moderating the CER-TFP relationship. The stronger positive moderating effect of ownership–control separation (*CER* × *Seperation*, *α_2_* = 0.005) in this specification suggests that in firms with greater separation, the adoption of entropy-weighted TFP might better capture the efficiency gains from CER, possibly because this measure is more sensitive to the heterogeneous ways different ownership structures transform environmental investments into productive outcomes.

Although the coefficient’s absolute value is relatively small, it precisely underscores the unique value and economic insight offered by the entropy weight method. This distinction is crucial for differentiating the economic implications of data-driven weighting (entropy weight method) from those of mathematically driven weighting (mean-value method, PCA). The moderating effect of ownership–control separation (*CER* × *Seperation*, *α_2_* = 0.005) is more pronounced in the *TFP-Entropy* model, indicating that the TFP index constructed via the entropy weight method exhibits higher identification sensitivity to heterogeneous efficiency paths. Unlike methods such as PCA method, which aim to extract common trends, the weight assignment in the entropy weight method is entirely driven by the degree of dispersion among the results of various TFP estimation methods (OLS, FE, LP, OP, and GMM). This dispersion reflects the heterogeneous mechanisms through which firms convert environmental investments into productive outcomes under different ownership structures. Consequently, a higher moderating coefficient suggests that firms with pronounced separation of ownership and control achieve more efficient heterogeneous transformation, and the entropy weight method, owing to its sensitivity to “differences”, is more capable of capturing this underlying economic reality. Given that the *TFP-Entropy* model exhibited a significantly higher goodness of fit (R^2^) and the coefficients for key variables were more statistically significant and better aligned with theoretical expectations, these findings provided direct empirical evidence supporting the superiority of the entropy weight method.

### 4.4. Results of Ownership Structure by the Method of Entropy

Table 4 demonstrates regression outcomes employing entropy-weighted ownership structures. Columns (1)–(2) feature *TFP-Mean* as the dependent variable, while columns (3)–(4) utilize *TFP-Entropy* in baseline specifications. We applied the entropy weighting method to compute three ownership variables: chairman shareholding ratio, manager shareholding ratio, and ownership–control separation ratio. This generated a comprehensive ownership structure variable (*OwnStrEnt*) and its interaction term with *CER* (*CER* × *OwnStrEnt*). Analysis reveals consistently positive *CER* coefficients significant at the 10% level. Notably, only column (4)’s interaction term achieved significance (*α_2_* = 0.021, *p* < 0.05). The consistency between entropy-weighted dependent and moderating variables indicates this method’s capacity to capture nonlinear relationships and distributional heterogeneity overlooked by conventional approaches, thereby better reflecting complex interdependencies in carbon-productivity linkages.

### 4.5. Results of Heterogeneity Analysis: Ownership Type

We subsequently conducted comparative analyses of CER’s impact on TFP across enterprise ownership types. Table 5 presents regression results with both *TFP-Mean* and *TFP-Entropy* as dependent variables. Columns (1) to (3) and columns (4) to (6) display the outcomes for state-owned enterprises (SOEs), privately owned enterprises (POEs), and foreign-owned enterprises (FOEs), respectively. *CER* coefficients demonstrate positive and statistically significant effects in POEs and FOEs, indicating that these enterprises prioritize TFP enhancement while advancing carbon reduction initiatives. Specifically, *CER* coefficients of 0.036 and 0.147 (column 2–3), 0.008 and 0.027 (column 5–6) reveal FOEs’ greater emphasis on TFP growth during decarbonization. Conversely, SOEs exhibit a statistically insignificant negative coefficient in columns (1) and (4), suggesting potential insufficient integration of TFP considerations in state-owned enterprises’ emission reduction strategies.

The observed heterogeneity in CER’s impact across ownership types reveals profound institutional and governance implications that extend beyond the initial statistical findings. The significantly stronger response from FOEs and POEs, compared to SOEs, can be attributed to fundamental differences in objective functions and incentive structures. FOEs, often subject to stringent global ESG standards and shareholder accountability, prioritize cost-efficient decarbonization that directly enhances operational efficiency. Conversely, SOEs may operate under a dual mandate that prioritizes social stability and employment over pure profit maximization, potentially treating carbon emission reduction as a regulatory compliance cost rather than a strategic investment in productivity. This aligns with the theoretical framework of the “resource-based view”, where the ability to transform environmental investment into competitive advantage is contingent upon a firm’s governance structure and managerial incentives. Consequently, the findings not only validate the core hypothesis that CER can drive TFP but also delineate its boundary condition: the effect is potent in market-oriented firms where financial performance is the primary goal, but muted in firms where corporate objectives are multifaceted and include socio-political mandates. This suggests that for decarbonization policies to universally enhance productivity, reforms aimed at hardening the budget constraints and aligning incentives within SOEs may be a prerequisite.

### 4.6. Results of Heterogeneity Analysis: Enterprise Scale

As illustrated in Table 6, this table presents heterogeneity regression analyses stratified by enterprise scale, employing both *TFP-Mean* and *TFP-Entropy* as dependent variables. Enterprises were stratified by total assets at the 50th percentile; the top 50% classified as large enterprises (columns 1 and 3), while the bottom 50% constituted small enterprises (columns 2 and 4). Columns (1) to (2) and columns (3) to (4) present the heterogeneity outcomes for large and small enterprises under the two respective dependent variable regressions. Positive *CER* coefficients in columns confirm CER’s beneficial impact on TFP. The coefficients for *CER* in the first two columns were 0.226 and 0.025, respectively, while those in the latter two columns were 0.042 and 0.006, indicating that large enterprises demonstrate greater responsiveness to TFP influences compared to small enterprises. Notably, only columns (2) and (4) achieve significance at the 5% level, indicating more pronounced TFP effects from emission reduction initiatives in smaller enterprises. Consequently, the statistically significant outcomes observed in smaller-scale enterprises corroborate Hypothesis 1.

The observed differential in *CER* coefficients between large and small enterprises, alongside their distinct significance patterns, reveals a profound scale-based heterogeneity in resource allocation and innovation capacity. Although the point estimate for large enterprises (0.042) is substantially higher than that for small ones (0.006), the differences likely stem from high fixed-cost investments in large-scale green technologies that exhibit long payback periods and greater outcome variance. Conversely, the statistically significant effect in small enterprises underscores their agility in implementing low-cost, incremental efficiency enhancements that yield immediate, measurable productivity gains within shorter operational cycles. This divergence aligns with the paradox of scale: large firms pursue transformative but high-risk innovations, while small firms excel in executing rapid-return projects. These findings refine rather than refute Hypothesis 1, confirming that CER enhances TFP across all scales, but through mechanistically distinct pathways. From a governance perspective, this implies that policy and management strategies must be scale-specific: large enterprises require incentives for long-term capital-intensive green innovation, whereas small enterprises need support for scalable, rapid-efficiency solutions.

### 4.7. Results of Heckman Two Stage

To mitigate endogeneity concerns, the Heckman two-stage model was implemented. During the initial stage, the dependent variable *TFP-Mean* and *TFP-Entropy* was transformed into a binary indicator—assigned 1 if exceeding their mean value and 0 otherwise. The probit model then computed the Inverse Mills Ratio (IMR) for each observation. Subsequently, the second-stage regression incorporated IMR as a control variable within the logit model. As presented in Table 7, columns (1) and (3) display first-stage results with a *CER* coefficient of 2.415 and 2.408. Columns (2) and (4) report second-stage outcomes, confirming statistically significant CER-TFP effects at the 5% level. The statistically insignificant IMR coefficient (*p* > 0.10) indicates negligible sample selection bias. Consequently, Heckman-adjusted estimates demonstrate robustness and align with baseline findings.

The statistically insignificant IMR coefficient across both TFP measures (*p* > 0.10) provides compelling evidence that our core pooled OLS estimates are unlikely to be substantially biased by sample selection, a concern inherent in studies focusing on firms’ voluntary environmental actions. This strengthens the credibility of our baseline findings. More importantly, the persistent significance of the *CER* coefficient in the second stage, even after controlling for potential selection, underscores that the positive relationship between CER and TFP is robust and not driven by unobserved factors influencing a firm’s decision to be a high or low TFP performance. This Heckman-adjusted result corroborates and deepens the support for Hypothesis 1. From a corporate governance perspective, the lack of significant selection bias suggests that the drive for productivity through decarbonization is not confined to firms with specific, unobserved traits but may be a more universal mechanism. This implies that boards and executives across a wide spectrum of firms can consider CER not merely as a regulatory compliance cost but as a general strategic lever for enhancing operational efficiency and long-term value, reinforcing the governance implications discussed in our main analysis.

### 4.8. Results of RDD

This study examines policy effects concerning China’s carbon emission trading pilot initiative. In 2011, aligned with the 12th Five-Year Plan’s mandate to ‘gradually establish a carbon emission trading market,’ China launched emissions trading pilots across seven regions: Beijing, Tianjin, Shanghai, Chongqing, Hubei, Guangdong, and Shenzhen, with carbon trading market operations commencing in 2013. We leveraged the province-staggered implementation of the pilot policy as an exogenous shock to construct models with temporal discontinuities. Comparative analysis between pilot regions (treatment group) and non-pilot regions (control group) assesses changes in the CER-TFP relationship pre- and post-implementation, thereby testing research hypotheses.

**Figure 1:**
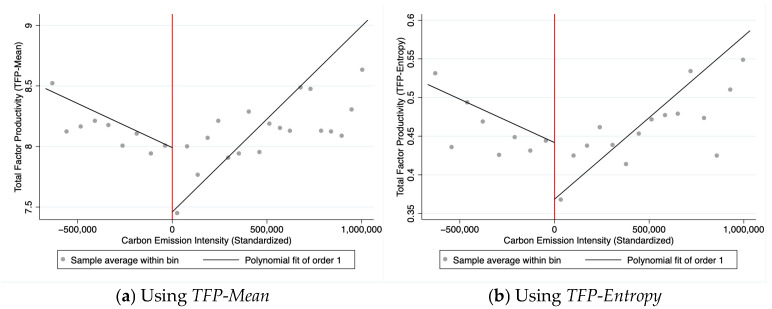
RDD plot for carbon trading pilot policy. Note: This figure displays regression discontinuity plots for the carbon emission pilot policy. The assignment variable is plotted on the x-axis, centered at the cutoff value. Outcome variables *TFP-Mean* and *TFP-Entropy* appear on the y-axis. Each point represents local polynomial smoothed averages within bandwidths. Vertical red lines mark cutoff points, while solid lines depict fitted regression curves on both sides of the threshold.

Figure 1a,b present RDD model results based on *TFP-Mean* and *TFP-Entropy* methodologies, respectively. Both figures employ the aforementioned pilot regions as policy effect benchmarks, with standardized carbon intensity on the x-axis and TFP metrics on the y-axis. At the discontinuity point (marked by red lines), both *TFP-Mean* and *TFP-Entropy* exhibit statistically significant jumps (Δ*TFP-Mean* ≈ 0.5; Δ*TFP-Entropy* ≈ 0.07), confirming that the pilot policy enhanced technical efficiency by strengthening emission constraints. Concurrent slope sign reversals observed before and after policy implementation reveal structural transformations in production induced by the policy, where high-emission enterprises’ expansion inhibits productivity while carbon reduction policies drive green technological upgrading. This reversal reinforces causal inference, supporting Hypothesis H1’s core logic: carbon trading mechanisms drive productivity innovation through cost internalization. Although both methodologies show similar trends, entropy-weighted results demonstrate superior model fit and policy effect identification.

The statistically significant jumps in both *TFP-Mean* and *TFP-Entropy* at the policy cutoff (discontinuity point), as revealed by our RDD analysis, provide strong evidence for a causal effect of the carbon emission trading pilot policy on enhancing technical efficiency. The observed magnitude of the jump (Δ*TFP-Mean* ≈ 0.5; Δ*TFP-Entropy* ≈ 0.07) signifies that the policy shock, which imposed stricter emission constraints, acted as a potent market-based instrument to internalize the environmental externality. This compelled firms in pilot regions to re-optimize their production processes, likely through adopting energy-saving technologies, improving managerial practices, and reallocating resources towards cleaner, more innovative activities. The concurrent reversal in the slope of the relationship between carbon emission intensity and TFP around the cutoff further underscores a fundamental structural transformation induced by the policy: it not only raised the productivity level but also altered the marginal returns to carbon efficiency. This aligns with and strongly reinforces the core logic of Hypothesis 1 proposed in our main analysis, demonstrating that the carbon trading mechanism drives productivity innovation through cost internalization and provides a credible quasi-experimental validation of the positive CER-TFP relationship we hypothesize.

### 4.9. Results of DID

Building upon prior regression discontinuity analyses of provincial carbon trading policy effects on TFP, this section examines temporal policy impacts. China ratified the Kyoto Protocol in 2005, actively participating in the Clean Development Mechanism (CDM). The national carbon emissions trading pilot program was initiated across seven provinces or cities in 2011, with regional carbon trading markets commencing operations starting in 2013. Fujian Province subsequently launched its carbon trading market as the eighth pilot region in 2016. Given the policy’s pilot implementation in 2011 and formal carbon trading market commencement in 2013, we designate 2013 as the de facto policy effectiveness year. Chinese enterprises consequently initiated CER projects incrementally in response to regulatory impacts.

**Figure 2:**
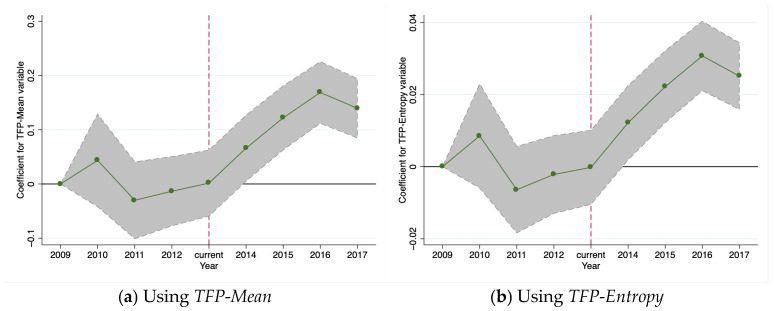
DID plot for carbon trading pilot policy. Note: This figure displays *TFP-Mean* and *TFP-Entropy* trajectories for treatment and control groups from four years pre-implementation to four years post-implementation. The policy enactment year (2013) is marked ‘current’. The sample contains 376 primary observations spanning 2009−2017. The green solid line traces *TFP-Mean* and *TFP-Entropy* coefficient dynamics, with the red dashed line indicating the 2013 policy implementation. The gray-shaded band represents the 90% confidence interval.

Empirically, the core identification prerequisite for the DID model is the parallel trends assumption. This assumption requires that, prior to the implementation of a policy intervention, the outcome variables of the treatment and control groups should exhibit common evolutionary trends. If the pre-treatment trends are inherently different, any observed divergence following the intervention becomes difficult to attribute solely to the policy effect, as it may merely reflect the continuation of pre-existing differentials. The pilot carbon emission trading policy examined in this study was implemented in two batches—the main group of cities initiated the program in 2013, followed by a limited expansion to specific cities in 2016. This staggered adoption of the policy may lead to heterogeneous or delayed effects across regions and time, thereby complicating the identification of the net policy impact. While the treatment and control groups might demonstrate distinct trends due to differing initial levels before the intervention, the parallel trends assumption can still be considered valid if these trend differentials remain stable and are not expected to alter their fundamental trajectory due to the intervention. In this study, we employed the methodology outlined by Beck et al. [84], which involved initially computing the pre-treatment averages and subsequently applying a demeaning procedure to the regression coefficients and confidence intervals across all time periods to effectively address potential pre-existing trends. Although such baseline differences may potentially violate the strict parallel trends assumption, it is noteworthy that its complete and perfect fulfillment is not always required [85]. Following the application of these methods to process the policy intervention data, parallel trend plots were generated as presented in Figure 2a,b. The parallel trends test conducted in this study provides supporting evidence for the validity of the underlying assumption.

We employed the DID model to assess policy effects by comparing pre-post changes in treatment groups (pilot regions) versus control groups (non-pilot regions). Figure 2a,b illustrates the temporal evolution of policy impacts on TFP, with 2013 as the implementation year and 2009 as the baseline. The study period spans 2009–2017, covering four pre-treatment and four post-treatment years. Pilot status determined group assignment, with pilot regions designated as the treatment group and non-pilot regions as the control group. The TFP coefficient trajectory reveals pre-policy oscillations near the zero baseline, transitioning to statistically significant positive values post-implementation. Particularly in 2014 (one year post-enactment), coefficients revealed significant positivity. This pattern confirms treatment-control group comparability while indicating reporting lag-induced delayed policy effects. Notably, pre-policy *TFP-Mean* and *TFP-Entropy* coefficients clustered tightly around zero with confidence intervals encompassing the zero baseline. Therefore, the core conclusions derived from both methodological approaches demonstrated consistency, substantially enhancing the reliability of the findings presented in this study. As illustrated in Figure 2a, the *TFP-Mean* coefficients exhibited relatively minor fluctuations alongside a generally smoother trend profile. In contrast, Figure 2b revealed that the *TFP-Entropy* metric displayed a more pronounced volatility in its trend dynamics; nevertheless, by reallocating weights to diminish the disproportionate impact of specific anomalous indicators, the resultant stability of the estimates proved superior. This outcome suggests that the entropy weighting approach effectively attenuates distortion caused by outliers through information entropy optimization, consequently fulfilling the parallel trends assumption under a more stringent criterion. Furthermore, comparative analysis between the two figures confirmed that the *TFP-Entropy* coefficients indeed manifested no statistically significant differences during the pre-policy trend period, thereby rendering the testing procedure more rigorous and the empirical results more robust. Collectively, these findings evidence significant policy-induced differentials during 2009–2017, further substantiating our hypotheses.

The evolution of TFP coefficients derived from our DID model offers compelling dynamic insights into the temporal impact of the carbon trading policy. The transition from pre-policy oscillations around zero to statistically significant positive values post-2013 confirms a causal policy effect that emerged after the carbon trading market’s commencement. The one-year lag observed for significant effects (appearing in 2014) is economically intuitive, reflecting the natural implementation delay as firms need time to adjust their capital stock, adopt new technologies, and reconfigure operations in response to new carbon pricing signals. The superior performance of the *TFP-Entropy* metric in satisfying the parallel trends assumption pre-treatment, compared to *TFP-Mean*, highlights its robustness in mitigating outlier distortion and providing a more reliable baseline for causal inference. The post-treatment divergence, although statistically insignificant between methodologies, consistently indicates a positive treatment effect. These findings from the DID design complement the RDD results by capturing the average treatment effect over time across all pilot regions, and are seamlessly integrated into our main narrative: they provide longitudinal evidence that the policy-induced CER efforts, through market mechanisms and regulatory compliance, lead to sustained TFP improvements by fostering long-term technological upgrading and efficiency gains.

## 5. Discussion and Implications

### 5.1. Discussion and Limitation

The implementation of carbon reduction policies presents a cost-productivity tradeoff for enterprises, wherein process innovation enhances production efficiency to facilitate industrial upgrading. This study uncovers the unique nexus between CER and TFP in China’s institutional context. Unlike developed economies, Chinese firms’ decarbonization relies on state-driven policy enforcement and ownership structure transformation. The strategic function of board chair ownership proves pivotal in policy-mandated emission control [86], while managerial ownership incentives reflect constraints in implementation efficiency [87]. The moderating effect of ownership–control separation reveals potential agency conflicts exacerbating resource misallocation [88]. These findings advocate a trinity governance synergy framework: securing technological investment continuity through chair shareholding, optimizing execution via managerial equity incentives, and constraining agency costs via control rights concentration. Current carbon market mechanisms urgently require incorporating ownership governance dimensions to assess corporate decarbonization potential [89]. In international markets, institutional investors leverage ESG engagement to catalyze emission innovations. Comparative analyses suggest variation in the CER-TFP marginal effects across these contexts compared to the effects observed in Chinese enterprise samples. China’s state-led pathway substitutes policy signals for shareholder monitoring, though efficacy depends on regional enforcement intensity. Ownership structures moderation effects exhibit institutional divergence [90,91]: International corporate board leverage market contracts, whereas Chinese board incentives face administrative constraints [11,92]. Progressive optimization of ownership governance not only mitigates financing costs but also elevates market participant assurance toward corporate prospects [69]. Consequently, developed economies should harness ownership catalysis in decarbonization, enhancing governance diversity within global carbon neutrality efforts. The cross-national comparison underscores institutional environments’ profound shaping of CER-TFP mechanisms.

This research pioneers a ‘governance-decarbonization’ coevolution framework bridging institutional and environmental economics. Policy-governance symbiosis emerges as critical, with industrial policy efficacy contingent upon micro-level governance structures. While international carbon markets utilize investor governance to lower transaction costs [93], China requires ‘policy-empowered governance’ to compensate for market mechanism deficiencies [94]. Thus, global carbon governance necessitates dual-track pathways: developed economies optimizing shareholder accountability mechanisms, and emerging economies designing policy toolkits. Future research could explore blockchain-enabled digital solutions to optimize corporate governance-carbon emissions alignment. This study’s limitations include constrained data availability due to non-mandatory carbon disclosure, impeding a comprehensive assessment of productivity impacts. Mandatory emission reporting would support more granular and statistically robust firm-level assessments while addressing potential endogenous concerns. Although mean-consolidated and entropy-weighted methods provided comparative insights, they only partially capture policy implementation effects, thus mandating refined methodological approaches for firm-level carbon emissions research. Future work may explore the impacts of regional policies or ESG disclosures on TFP.

### 5.2. Implications and Future Research

The empirically observed positive CER-TFP relationship, coupled with the heterogeneous moderation effects of ownership structures, provides a robust foundation for formulating targeted and effective decarbonization policies. Specifically, the significantly stronger TFP responsiveness to CER initiatives observed in POEs, FOEs, and small-scale firms suggests that policy incentives such as tax benefits, green technology subsidies, or streamlined carbon market access could be most effective when preferentially directed towards these entities to maximize aggregate productivity gains. Conversely, the muted or statistically insignificant response in SOEs and certain large enterprises underscores a critical need for governance-centric policy interventions. For these firms, policies should mandate and incentivize the deep integration of carbon emission reduction targets into executive performance evaluation systems and corporate strategic planning, thereby aligning managerial incentives with long-term environmental and productivity goals. Furthermore, the superior performance of the entropy-weighted TFP metric in capturing the nuanced effects underscores the value of adopting sophisticated measurement tools in policy design and evaluation. Policymakers are advised to incorporate such multi-faceted productivity assessments into the carbon market’s regulatory framework and enterprise environmental performance ratings. This would enable a more accurate identification of firms that are genuinely achieving green productivity synergies, ensuring that policy support and market rewards are allocated efficiently to accelerate China’s transition to a high-quality, low-carbon economy.

This study yields significant implications for practitioners, investors, and policymakers. First, the positive CER-TFP relationship reveals the environmental strategy’s productivity value, yet ownership structure’s moderating effects necessitate governance logic reconstruction. Empirical analyses confirm that corporate CER initiatives enhance TFP growth. Consequently, executives should proactively formulate emission reduction policies aligned with national regulations while refining environmental production standards. Furthermore, monitoring internal ownership dynamics enables structural optimization that facilitates both carbon compliance and TFP gains. Future practice requires a trinity governance synergy: leveraging chair shareholding for long-term technological investment, managerial shareholding for strengthening emission execution, and control rights concentration to reduce agency costs. Enterprises must develop “governance-emission adaptation models” to dynamically optimize equity incentives, integrate carbon metrics into executive performance systems, and achieve co-evolution of environmental and productivity goals. For investors, proactive engagement with national pilot policies enables timely production facility upgrades. And ownership moderation effects further provide an ESG evaluation framework requiring three signal discernments: (1) high chair shareholding firms generate “green innovation premiums” under carbon constraints due to strategic stability; (2) manager shareholding ratios demand scrutiny to prevent incentive-driven carbon arbitrage; (3) excessive ownership–control separation risks emission resource misallocation, potentially triggering valuation discounts during policy tightening. Investors should also collaborate with research institutions to construct a “low-carbon governance database” tracking marginal contributions of ownership reforms to carbon emissions and productivity. Policymakers must establish industry dialogs to identify manufacturing decarbonization challenges. Implementation should feature categorical enterprise governance, batched policy trials, and ownership optimization guidance to reduce resource misallocation and agency conflicts. Incorporating governance indicators into carbon market assessments—such as mandating disclosure, strategic executive-supervisory governance structures—is critical. Future mechanisms require “policy-governance” linkages and dual-dimension “emission-governance” evaluations to advance national carbon market reforms.

## 6. Conclusions

Using 2009–2023 data from China’s non-financial listed firms, pooled OLS models examined CER’s impact on TFP. Results confirmed sustained TFP improvements post CER implementation. Empirical analysis through chairman shareholding, manager shareholding, and ownership–control separation ratios demonstrates the ownership structure’s moderating role. Mean-consolidated and entropy-weighted methodological comparisons validate approach superiority. Additionally, private, foreign-owned, and smaller enterprises exhibit heightened TFP sensitivity to CER. Despite batched policy implementation lags, significant treatment effects on TFP materialize. Collectively, these findings substantiate CER’s positive TFP impact and underlying mechanisms in China’s listed firms.

## Figures and Tables

**Table 1:** Descriptive statistics.

Variable	Obs	Mean	Std. Dev.	Min	Max
*TFP-Mean*	18,866	8.027	0.965	5.8	11.2
*TFP-Entropy*	18,866	0.406	0.181	0	1
*CER*	1190	9.149	2.996	−0.511	17.292
*CShare*	26,186	11.157	15.09	0	56.83
*MShare*	25,864	7.016	12.631	0	53.49
*Seperation*	25,968	4.299	6.964	0	27.171
*Age*	23,276	9.452	7.025	1	28
*Ownership*	26,960	1.834	0.558	1	3
*TAssets*	26,960	21.915	1.214	19.51	25.805
*RDExp*	12,959	17.977	1.392	13.972	21.63
*OReve*	26,689	21.172	1.347	18.183	25.061
*Employee*	26,942	3533.918	6108.52	87	42,239
*Leve*	27,953	1.256	0.781	0.415	6.436

Note: The sample comprised 1123 firm-year observations from 2009 to 2023. The main variables were winsorized at the 1st and 99th percentiles. See Appendix A for the definitions of the variables.

**Table 2:** Baseline regression of *TFP-Mean*.

	(1)	(2)	(3)	(4)
	Model 1	Model 2	Model 3	Model 4
*CER*	0.037 **	0.045 **	0.059 **	0.053 **
*CShare*		−0.003 *		
*CER* × *CShare*		−0.002 **		
*MShare*			0.003 *	
*CER* × *MShare*			0.003 *	
*Seperation*				0.034
*CER* × *Seperation*				0.001 **
Cons	YES	YES	YES	YES
Adj. R^2^	0.209	0.250	0.244	0.237
N	1121	1121	1121	1121

Note: Regression results of Equations (1), (4), (5), and (6). The sample comprises 1123 firm-year observations from 2009 to 2023. The results are from a pooled OLS model regression with *TFP-Mean* as a dependent variable. * and ** denote the significance at the 10%, 5%, levels, respectively. See Appendix A for the definitions of the variables.

**Table 3:** Baseline regression of *TFP-Entropy*.

	**(1)**	**(2)**	**(3)**	**(4)**
	**Model 1**	**Model 2**	**Model 3**	**Model 4**
*CER*	0.005 ***	0.002	0.004	0.004 **
*CShare*		−0.001		
*CER* × *CShare*		−0.001 *		
*MShare*			0.000	
*CER* × *MShare*			0.002 **	
*Separation*				0.003
*CER* × *Separation*				0.005 **
Cons	YES	YES	YES	YES
Adj. R^2^	0.332	0.374	0.356	0.303
N	1123	1123	1123	1123

Note: Regression results of Equations (1), (4), (5), and (6). The sample comprises 1123 firm-year observations from 2009 to 2023. The results are from a pooled OLS model regression with *TFP-Entropy* as a dependent variable. *, **, and *** denote the significance at the 10%, 5%, and 1% levels, respectively. See Appendix A for the definitions of the variables.

**Table 4:** Results of ownership structure by the method of entropy.

	(1)	(2)	(3)	(4)
	*TFP-Mean*	*TFP-Mean*	*TFP-Entropy*	*TFP-Entropy*
*CER*	0.024 **	0.011 *	0.005 ***	0.003 *
*OwnStrEnt*	−0.291	−0.412	−0.046	−0.070
*CER* × *OwnStrEnt*		0.011		0.021 **
Cons	YES	YES	YES	YES
Adj. R^2^	0.413	0.414	0.496	0.497
N	1123	1123	1123	1123

Note: Regression results of Equations (2) and (3). The sample comprises 1123 firm-year observations from 2009 to 2023. The results are from a pooled OLS model regression with *TFP-Mean* and *TFP-Entropy* as dependent variable, respectively. *, **, and *** denote the significance at the 10%, 5%, and 1% levels, respectively. See Appendix A for the definitions of the variables.

**Table 5:** Results of heterogeneity analysis: ownership type.

	(1)	(2)	(3)	(4)	(5)	(6)
	*TFP-Mean*			*TFP-Entropy*		
	SOEs	POEs	FOEs	SOEs	POEs	FOEs
*CER*	−0.008	0.036 **	0.147 *	−0.002	0.008 **	0.027 *
Cons	YES	YES	YES	YES	YES	YES
Adj. R^2^	0.372	0.284	0.359	0.265	0.234	0.327
N	270	411	75	270	411	75

Note: Regression results of Equation (1), specifically for different levels of firms. Firms are classified as SOEs, POEs, and FOEs based on ownership type. The sample comprises 1123 firm-year observations from 2009 to 2023. The results are from a pooled OLS model regression with *TFP-Mean* and *TFP-Entropy* as dependent variables. * and ** denote the significance at the 10%, 5%, levels, respectively. See Appendix A for the definitions of the variables.

**Table 6:** Results of heterogeneity analysis: enterprise scale.

	(1)	(2)	(3)	(4)
	*TFP-Mean*		*TFP-Entropy*	
	Large	Small	Large	Small
*CER*	0.226	0.025 **	0.042	0.006 **
Cons	YES	YES	YES	YES
Adj. R^2^	0.347	0.314	0.328	0.364
N	582	319	582	319

Note: Regression results of Equation (1), specifically for different levels of firms. Firms are classified as large and small based on total assets. The sample comprises 1123 firm-year observations from 2009 to 2023. The results are from a pooled OLS model regression with *TFP-Mean* and *TFP-Entropy* as dependent variables. ** denote the significance at the 5% levels, respectively. See Appendix A for the definitions of the variables.

**Table 7:** Results of the Heckman two-stage process.

	(1)	(2)	(3)	(4)
	*TFP-Mean*		*TFP-Entropy*	
	Stage 1	Stage 2	Stage 1	Stage 2
*CER*	2.415	0.021 **	2.408	0.005 **
Inverse Mills Ratio		−0.002		−0.001
Cons	YES	YES	YES	YES
Adj. R^2^		0.215		0.203
N	1123	356	1123	356

Note: Heckman two-stage results of Equation (1). The sample comprises 1123 firm-year observations from 2009 to 2023. The results are from probit and OLS model regression with *TFP-Mean* and *TFP-Entropy* as dependent variables. ** denote the significance at the 5% levels, respectively. See Appendix A for the definitions of the variables.

## Data Availability

The data presented in this study are openly available in the China Stock Market and Accounting Research (CSMAR) Database and Chinese Industrial Enterprises Database.

## Appendix A

**Table A1:** Definitions of variables.

Variable Type	Name of Variable	Variable Symbol	Variable Definition
Dependent variable	Total factor productivity	*TFP*	Measured as the ratio of aggregate output (e.g., GDP) to aggregate inputs. (Unit: dimensionless)
Independent variable	Carbon emissions reduction	*CER*	The reduction in carbon emissions of a firm (Unit: tons/year)
Moderator variable	Ownership structure	*OwnStrEnt*	Ownership structure by the method of entropy
	Chairman shareholding ratio	*CShare*	The percentage of a company’s shares that the chairman owns (Unit: %)
	Manager shareholding ratio	*MShare*	The percentage of a company’s shares that the manager owns (Unit: %)
	The separation ratio of ownership and control	*Separation*	The degree of separation between capital ownership and capital operation rights in a firm (Unit: %)
Control variable	Listing age	*Age*	The length of time a firm has been listed (Unit: year)
	Ownership type	*Ownership*	The type of ownership: (1) state-owned enterprises, (2) private-owned enterprises, (3) foreign-owned enterprise
	Total assets	*TAssets*	The natural logarithm of total assets of a firm (Unit of total assets: yuan)
	RDExp	*RDExp*	The natural logarithm of research and development expense (Unit of R&D expense: yuan)
	Operating Revenue	*OReve*	The natural logarithm of operating revenue of a firm (Unit of operating revenue: yuan)
	Number of employees	*Employee*	Total number of employees of a firm (Unit: persons)
	Total leverage	*Leve*	The effect of operating and financial leverage on a company’s earnings per share in relation to its sales revenue. (Unit: %)
